# Serial-Omics and Molecular Function Study Provide Novel Insight into Cucumber Variety Improvement

**DOI:** 10.3390/plants11121609

**Published:** 2022-06-20

**Authors:** Danni Han, Xiaojun Ma, Lei Zhang, Shizhong Zhang, Qinghua Sun, Pan Li, Jing Shu, Yanting Zhao

**Affiliations:** 1 Institute of Vegetables, Zhejiang Academy of Agricultural Sciences, Hangzhou 310021, China; hdn17861506019@163.com; 2 State Key Laboratory of Crop Biology, College of Life Science, Shandong Agricultural University, Taian 271018, China; zhanganqi2002@163.com (L.Z.); shizhong@sdau.edu.cn (S.Z.); qhsun@sdau.edu.cn (Q.S.); 3 College of Forestry Engineering, Shandong Agriculture and Engineering University, Jinan 250100, China; mxjun@163.com; 4 School of Pharmacy, Liaocheng University, Liaocheng 252000, China; lipan@lcu.edu.cn

**Keywords:** cucumbers, omics, molecular marker, breeding, trait regulation

## Abstract

Cucumbers are rich in vitamins and minerals. The cucumber has recently become one of China’s main vegetable crops. More specifically, the adjustment of the Chinese agricultural industry’s structure and rapid economic development have resulted in increases in the planting area allocated to Chinese cucumber varieties and in the number of Chinese cucumber varieties. After complete sequencing of the “Chinese long” genome, the transcriptome, proteome, and metabolome were obtained. Cucumber has a small genome and short growing cycle, and these traits are conducive to the application of molecular breeding techniques for improving fruit quality. Here, we review the developments and applications of molecular markers and genetic maps for cucumber breeding and introduce the functions of gene families from the perspective of genomics, including fruit development and quality, hormone response, resistance to abiotic stress, epitomizing the development of other omics, and relationships among functions.

## 1. Introduction

Cucumber (*Cucumis sativus* L.; Cucurbitaceae) produces fruits that are both nutrient- and flavor-rich and that are consumed worldwide. Cucumber’s small genome and short growing cycle facilitate the application of molecular breeding techniques for improving fruit quality. The development of genomics is beneficial to understanding and mastering cucumber’s physiological traits, and the growth and development laws of cucumber at the molecular level for better research and utilization. This review summarizes the development and application of genetic maps, molecular markers, and functional gene annotation for cucumber breeding. It also discusses the development of cucumber omics, including genomics, transcriptomics, proteomics, and metabolomics.

## 2. Germplasm Resources and Molecular Markers

### 2.1. Origin and Varieties

Cucumber is an annual vine that originated in the rainforests of the southern Himalayan foothills [1]. The fruit is blue–green or light green and is adorned with relatively small and soft spines. Humans opened the market to cucumbers over 5000 years ago, and as early as 3000 years ago, the cucumber was planted in India, from which it was dispersed globally [2], eventually becoming one of the most important vegetable crops worldwide [3].

### 2.2. Molecular Markers and Genetic Maps

Molecular genetic maps facilitate positional cloning, whole-genome sequencing, and molecular breeding using breeder-friendly molecular markers. Molecular markers are new technologies based on morphology, cytology, and biochemical markers. With the rapid development of molecular biology, this technology has been used extensively in crop breeding. Breeders have used restriction fragment length polymorphism (RFLPs), random amplified polymorphic DNA (RAPD), simple sequence repeats (SSRs), and other markers to construct genetic maps, which can then be used to map crop traits [3]. The first genetic map of cucumber separated 13 morphology- and disease resistance-related genes into four linkage groups [4], and in 1994, Kennard [5] constructed a cucumber genetic map that contained 58 markers. Using 77 RAPD markers and three morphology markers, Serken et al. [6] constructed a genetic map of quantitative trait loci (QTLs) and grouped the markers into nine linkage groups. One hundred F3 families were used to identify sex expression and other QTL traits. Although recombinant inbred line (RIL) mapping can circumvent many of the shortcomings of F_2_ mapping, it was not until 2000 that a cucumber linkage map, which included 353 markers, was generated using RILs [7]. After one year, amplified fragment length polymorphism (AFLP) markers were added to existing narrow- and wide-based maps, including morphological characteristics and disease resistance loci, isoenzymes, RFLP, RAPD, and AFLP, which increased the combined map scores to 255 and 197 markers [8]. Simple sequence repeat markers are reproducible, multiple allelic properties, codominantly inherited, relatively abundant, and widely distributed among genomes, and thus are powerful tools for evaluating genetic variation. Sixty one *Cucumis* SSR markers were developed, and tens of the markers were evaluated for length polymorphisms among 11 cucumber genotypes [9]. Expressed sequence tag (EST)-derived SSR markers have many inherent advantages over genomic SSR markers because they have higher transferability between related species and can be developed at lower costs. As EST-SSRs are derived from transcripts, they are more valuable for genetic diversity analysis, marker-assisted selection, and comparative mapping. A total of 3627 unigenes was generated by assembling 5563 cucumber ESTs and gene sequences from GenBank, and 479 SSR loci were identified [10]. A cucumber genetic map, including 234 SSR markers in seven linkage groups, was constructed using a population of 179 F_2_ individuals from a cross of “PI183967” and the Northern Chinese-type inbred line, “931” [11]. After a year, a genetic map was developed using 248 microsatellite loci and 148 RILs that were derived from a cross between two inbred lines (9110Gt and 9930). The map revealed that four fruit epidermis-related genes were tightly linked on chromosome 5 and that three others were located at different places on chromosome 6 [12]. By comparing 13 genomic microsatellites (gSSRs) and 16 EST-SSR markers to estimate the genetic diversity of 29 cucumber accessions, an independent subpopulation was identified, including five accessions resistant to downy mildew [13].

The most important economic traits of cucumber are its quantitative traits. Genetic maps can be constructed to locate quantitative trait loci and then identify linked molecular markers. Finally, they can be applied to breeding programs. Quantitative trait locus mapping and analysis were conducted for cucumber fructification characteristics using an SSR linkage map that was constructed using 148 F_9_ RILs from a narrow cross between “9110Gt” and “9930Gt.” In this map, 32 QTLs were associated with 14 fructification characteristics, which provided insight into the genetic mechanisms underlying cucumber fruit traits [14]. Based on the “GY14” × “PI 183,967” map from the inter-subspecies cross and the extended “S94” × “S06” map from intra-subspecies hybridization, a high-density consensus map with high marker density and ordered markers was constructed. The resulting map included 1369 loci [15]. Inbred lines “1507” and “1508” were used as the experimental materials, and genetic analysis indicated that white peel coloration was dominated by a recessive nuclear gene. Bulked segregant analysis (BSA) and SSR technology evaluated the linkages of 14 SSR markers, which were subsequently associated with 500 candidate genes [16]. A high-density single nucleotide polymorphism (SNP) map of cucumber, which was established using specific-length amplified fragment sequencing (SLAF-seq), contained 1800 SNPs and nine predicted fruit length and weight QTLs [17]. The QTL mapping of cucumber fruit size using RILs from a cross between two inbred lines (“Gy14” and “9930”) resulted in the detection of 12 QTLs related to fruit elongation and radial growth [18]. Fifty-one pairs of SSR primers were used to analyze the genetic diversity of 42 cucumber varieties and ultimately detected 129 polymorphic loci, of which 95.6% were polymorphic [19]. Meanwhile, QTL mapping and QTL-seq for cucumber fruit length revealed eight QTLs for immature and mature fruit length, thereby providing a reference for the fine mapping of fruit length-related loci [20]. A genetic linkage map of 133 SSR and 9 insertion/deletion markers on 7 chromosomes was constructed from an F_2_ population of a cross between “EC1” and “8419 s-1“ [21]. One hundred and four cucumber genotypes were evaluated using 23 pairs of SSR primers, and 67 alleles were identified; there was a mean of 2.91 alleles per locus [22]. Based on 182 cucumber resequencing datasets, DNA fingerprints were established for 261 cucumber varieties through target SNP-seq, which found that 163 perfect SNPs came from 4,612,350 SNPs [23].

## 3. Serial-Omics and Database

### 3.1. Transcriptome Research of Cucumber

The plant transcriptome contains information on plant growth, development, and morphogenesis at the level of gene expression. Plant transcriptomes vary both temporally and spatially, which reflects the important role of the transcriptome in plant growth and development. The analysis of plant transcriptomes during fungal infections is an important strategy for accelerating crop research. For example, full transcriptome analysis of cucumber challenged with *Pseudoperonospora cubensis* revealed the differential expression of 15,286 genes [24]. Meanwhile, the transcriptome analysis of both resistant and susceptible cucumber varieties infected with obligate oomycete pathogens revealed significant differentially expressed genes in the plants’ leaves [25]. Ninety upregulated and 360 down-regulated genes were detected in cucumber roots after infection with *Fusarium oxysporum* f. sp. *cucumerinum* (Foc) [26], and in another study, the application of exogenous ethephon was shown to contribute to the viral infection resistance of cucumber seedlings [27]. Both the cytological and transcriptomic responses of cucumber to *Pseudomonas syringae* pv. *lachrymans* (*Psl*) were characterized to elucidate the mechanism underlying cucumber resistance to bacterial angular leaf spot disease [28].

Cucumber transcriptome analysis lays the foundation for improving fruit quality. The transcription of cell division genes is reportedly greater in parthenocarpic fruits. Among them, the transcription analysis of 14 predicted genes revealed crosstalk between indole-3-acetic acid (IAA), cytokinin (CTK), and gibberellin (GA) in the process of parthenocarpy fruit setting [29]. The cucumber mutant allele *short fruit 1*, which is associated with a short-fruit phenotype caused by reduced cell number, may also be involved in the syntheses and signal transmission mechanisms of these three hormones [30]. Based on transcriptome analysis, authors have suggested that microtubules, cell cycle-related genes, and transcription factors are associated with the formation of cucumber fruit [31].

Rootstocks can reduce cucumber quality. Transcriptomic analysis of cucumber fruits grafted onto different rootstocks revealed that 10 candidate genes were associated with sugar metabolism and linoleic acid and amino acid biosynthesis in grafted cucumber plants [32]. A predictive regulatory network for anthocyanin biosynthesis has been established to explore the molecular mechanism that regulates cucumber skin color development, and this work laid the foundation for cucumber breeding and the improvement of fruit skin color [33]. Furthermore, the transcriptome dataset provides a wide range of sequence resources for further research on cucumbers at the molecular level, in terms of drought, heat, toxin, salt, and waterlogging stresses [34,35,36,37,38].

### 3.2. Proteome Research of Cucumber

Proteomics is used to elucidate the proteins involved in specific physiological processes at the biological and cellular levels and to investigate changes in broad-scale protein expression, functions performed, post-translational modification status, and protein–protein interactions. Currently, cucumber proteomics has flourished in various fields. A proteomic study of scion-rootstock graft revealed 50 proteins that were differentially expressed and that those proteins were involved in a wide range of functions, including photosynthesis, carbohydrate metabolism, energy metabolism, and protein metabolism [39]. Cluster analysis showed that 41 parthenocarpy-related, differentially expressed proteins were screened in cytokinin-induced and naturally occurring parthenocarpic fruits, which confirmed that hormone-insensitive proteins can manipulate the mechanism of hormone-independent parthenocarpy [40]. The application of a plant growth-promoting *Trichoderma* strain (*T. longibrachiatum* “H9”) to cucumber roots resulted in the upregulation of genes and proteins that were mainly involved in defense/stress processes, secondary metabolism, phytohormone synthesis, and signal transduction [41]. In another study, the application of *T. guizhouensis* “NJAU 4742” to cucumber roots in a hydroponic system resulted in the identification of a high-abundance protein that regulates the shikimate pathway [42]. One hundred peptides were detected in the proteomes of downy mildew-resistant and -susceptible cucumber varieties (“ZJ” and “SDG”, respectively) that were infected by *P. cubensis*, which indicated that the induced accumulation of terpenoids contributes to cucumber resistance to *P. cubensis* infection [43].

In cucumber, proteomic analysis has been applied to studies of seedling root metabolism [44], the salinity mechanism in phloem [45], root putrescine responses [46], seeds protected with exogenous spermidine [47], and H_2_S regulation during salt stress [37]. One study analyzed the proteomes of Fe-starved roots and discovered that Fe deficiency affects metabolic processes, especially the increases in glycolytic flux and anaerobic metabolism to maintain dynamic [48]. Under hypoxic stress, proteins involved in a variety of metabolic pathways and defense mechanisms were differentially expressed. Cucumber uses antioxidant enzymes and acyl-[acyl-carrier-protein] desaturases to prevent reactive oxygen species from damaging the structure of cells [49]. In addition, increasing exogenous calcium levels results in the upregulation of enzymes related to metabolic and physiological systems, such as glycolysis, to moderate hypoxic stress [50]. Proteomics technology was used to compare the waterlogging tolerant and sensitive cucumber strains “Zaoer-N” and “Pepino,” respectively, under waterlogging stress. The maintenance of glycolysis played a significant role in alleviating hypoxic stress [51]. Exogenous Se reduces cucumber growth inhibition by affecting a variety of metabolic pathways, improving induced Fv/Fm ratio reduction, and ameliorating photosynthesis [52]. A comparative proteomics analysis study that was performed to improve the current understanding of ABA and H_2_O_2_ mediated regulation of adventitious root growth under drought stress suggested that H_2_O_2_ affects ABA-induced adventitious root growth under drought stress by regulating both photosynthesis-related and stress-defense-related proteins [53]. Furthermore, the accumulation of CO_2_ has been demonstrated to mitigate drought stress damage by reducing toxic substances [54].

### 3.3. Metabolome Research of Cucumber

Metabolomics provides a reliable strategy for investigating the remodeling of plant tissues and metabolites under different environmental conditions. The integration of metabolome, genome, transcriptome, proteome, and phenome studies is critical for plant breeding and for studying plant molecular mechanisms. The cucumber cultigen “Vlaspik” was found to be resistant to *Phytophthora capsici* at 16 d after pollination. Metabolomic screening of “Vlaspik” at 16 d after pollination for 113 ions revealed that two of the more abundant ions were glycosylated norterpene esters [55]. The dynamic metabolic profile of cucumber fruit contained 38 metabolites. Concentrations of several amino acids, carbohydrates, and flavonoids increased with the progression of fruit development [56]. The exogenous application of 2,4-dichlorophenoxyacetic acid can affect metabolite levels, mainly by affecting methionine metabolism, the citric acid cycle, and flavonoid metabolism [57]. The dark- and light-green-skinned cucumber genotypes “Lv” and “Bai”, respectively, accumulate different levels of key anthocyanins and flavanols in the skins of their fruits [33]. Iron (Fe) treatment significantly affects levels of serine, succinic, and fumaric acids under aluminum (Al) stress, which suggests that the chelation of Fe contributes to Al stress tolerance by balancing the Fe and Al contents of the xylem sap. Both Fe and Si can help plants exclude Al under acidic conditions [58]. Excess copper can disrupt carbohydrate metabolism, and antioxidant and defense mechanisms. Moreover, polyphenol metabolomics has revealed decreased flavonoid levels [59]. Levels of 26 differential metabolites involved in the metabolism of alanine, aspartic acid, and glutamate were evaluated using a non-targeted metabolomics approach to investigate the effects of atmospheric CO_2_ level and CO_2_ fertilization on drought stress [60].

### 3.4. Information Resources for Cucumber Research

Owing to the rapid development of sequencing technologies, high-quality reference genome sequences for cucumber have been generated and released. Numerous databases have been created to store, mine, analyze, and disseminate vast transcriptomic and genetic datasets and to provide a central portal for research and breeding communities. The genomics and functional genomics databases specially constructed for the Cucurbitaceae, including cucumber, include the Cucurbit Genomics Database (CuGenDB, http://www.cucurbitgenomics.org/, accessed on 15 December 2021) [61] and the cucumber alternative splicing (CuAS) database (http://cmb.bnu.edu.cn/alt_iso/index.Php, accessed on 20 December 2021) [62].

CuGenDB is a dynamic database that integrates the rich genomic and genetic resources of cucurbits. To date, the database includes 10 cucurbit genome sequences, 265,334 protein-coding gene sequences, 1.74 million ESTs, and 21 maps. The database also provides various query, visualization, and analysis tools for genomics and breeding studies [61]. Meanwhile, the CuAS database includes AS transcripts from cucumber and annotations that include genomic information, AS events, isoform functions, isoform features, and tissue-specific splicing events. CuAS can be used to explore the relationships between functional features and predicted AS transcripts [62].

Furthermore, Phytozome (https://phytozome-next.jgi.doe.gov/, accessed on 15 December 2021) [63], Gramene (https://www.gramene.org/, accessed on 15 December 2021) [64], and PlantGDB (http://www.plantgdb.org/, accessed on 21 December 2021) [65], as multiple genome comparison databases, can also be used for the analyzing and comparing cucumber omics data.

### 3.5. Salt Tolerance PPI Network of Cucumber

**Figure 1:**
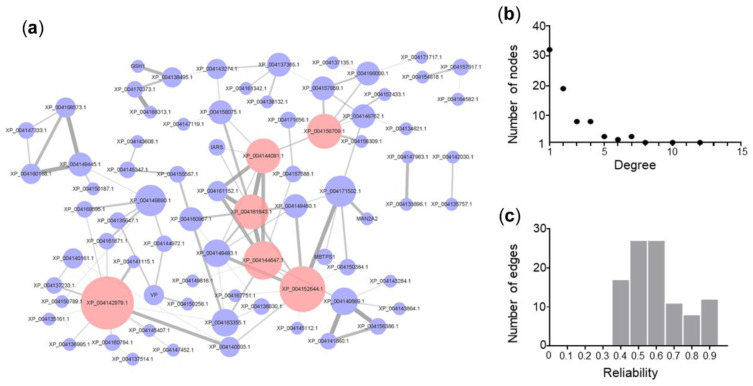
Protein–protein interaction (PPI) network of salt tolerance in cucumber. (**a**) PPI network. (**b**) Distribution of PPI network node degree. (**c**) Distribution of PPI network edge reliability.

The assembly of biological networks has been improved by the discovery of omics data. Protein–protein interaction (PPI) networks are now some of the most important and widely studied networks, thereby advancing the current understanding of potential cellular processes [66,67]. Protein–protein interaction modules are groups of proteins involved in specific functions, such protein complexes, physiological pathways, or regulatory systems [68]. Soil salinization has caused serious damage to plant growth and crop yields worldwide. Using CuGenDB and Cytoscape software [69], we identified 78 proteins that are involved in salt resistance and generated a PPI network model. The top six hub nodes are XP_004142979.1, XP_004152644.1, XP_004144647.1, XP_004144081.1, XP_004161843.1, and XP_004156709.1 (Figure 1a); and the relationship between node number and degree suggests a maximum degree of 32 (Figure 1b), whereas the relationship between edge number and reliability suggests that ~30% of edges have reliability values of >70% (Figure 1c).

## 4. Gene Function Analysis and Trait Regulation

With the development of high-throughput sequencing technology and whole-genome sequencing [70], data on cucumber stress resistance, plant hormones, and fruit quality have gradually increased. The progress of molecular cucumber breeding requires the analysis of sequencing data related to commercial traits [71]. Analysis of the Gy14 cucumber genome resulted in the identification of 112,073 perfect repeats, which account for 0.9% of the assembled Gy14 genome [72]. A genome-wide genetic variation map has been identified by more than 360 loci, which were generated by deep sequencing of 115 cucumber lines, laying the foundation for genome-wide design and breeding [73].

### 4.1. Functional Genes That Regulate Development and Quality

Many genes related to cucumber fruit development and quality, including spine and skin color [74,75,76,77,78,79], locule formation [80], fruit length [20,81], spine density and development [82,83], and rind patterns (i.e., striping) [84], have been identified and studied. Cucumber varieties with strong parthenocarpic tendencies exhibit stable seed setting rates, thicker fruit flesh, smaller fleshy cavities, and better flavor than conventionally pollinated fruits. A cucumber linkage genetic map was preliminarily located on the main QTL for cucumber parthenocarpy and constructed based on 90 SSR markers. A MYB family transcription factor was predicted to play a critical role in the regulation of parthenocarpy [85].

### 4.2. Functional Genes That Regulate Hormone Responses

As important plant hormones, IAA, CTK, brassinosteroid (BR), GA, and ethylene play important roles in regulating physiological processes. A genome-wide survey of cucumber revealed 16 auxin-response factor (ARF) genes, 27 auxin/indole acetic acid (Aux/IAA) genes, 10 gretchen hagen 3 (GH3) genes, 61 small auxin-up mRNA (SAUR) genes, and 39 lateral organ boundary (LBD) genes that were predicted to regulate various growth and development mechanisms [86]. The *tuberculate fruit gene* (*Tu*), which was obtained from map-based cloning, promotes the biosynthesis of CTK in fruit warts, which can be identified in 38 wart-like strains [87]. Dwarfism caused by BR deficiency can improve plant yield by manipulating the plant height. One study reported that the dwarf cucumber mutant *super compact-2* (*scp-2*), whose condition is caused by mutations in *cucumber deetiolated2* (*CsDET2*), exhibits impaired BR synthesis [88]. Twenty-seven putative teosinte branched1/cycloidea/proliferating cell factor (TCP) genes have been identified and induced by GA and ethylene treatments [89].

### 4.3. Functional Genes That Resist Abiotic Stress

Several gene families related to abiotic stress resistance have been identified and investigated. These include WAX [90], YUCCA (YUC) [91], stachyose synthase (STS) [92], plant glycine-rich RNA-binding protein (GR-RBP) [93], basic pentacysteine (BPC) [94], WRKY [95], trehalose-6-phosphate synthase (TPS) [96], tubby-like protein (TLP) [97], and zinc finger-homeodomain (ZF-HD) family genes [98]. In addition to providing a valuable basis for functional research, related studies have analyzed the different stress responses of these genes and the physiological regulatory pathways involved. Fourteen MAPK genes, six MAPKK genes, and 59 MAPKKK genes were identified; and most of these genes are differentially expressed under high temperature, low temperature, drought, and *P. cubensis*-induced stress [99]. One study reported that golden2-like proteins (GLKs) contribute to the regulation of cucumber mosaic virus tolerance in *Arabidopsis* [100]. Cas9/subgenomic RNA (sgRNA) technology has been used to generate recessive inactivation of *eukaryotic translation initiation factor 4E* (*eIF4E*) gene. This research suggests that *eIF4E* inhibition promotes resistance against Cucumber vein yellowing virus [101]. In the cucumber drought stress regulatory pathway, cucumber activating factor1 (CsATAF1) is a positive regulator that can reduce the accumulation of reactive oxygen species (ROS) [102]. Information retrieved from genome assembly may provide important clues about various molecular aspects of plants. Forty homeodomain-leucine zipper (HDZ) genes were detected in the cucumber genome database and determined to play roles in a variety of abiotic stress and powdery mildew stress resistance regulatory mechanisms [103].

## 5. Application of Omics Techniques and Molecular Markers to Breeding

In recent years, the development of novel cultivars and mining of important agronomic traits have become increasingly inseparable from the applications of molecular markers. This provides a broad-based and effective approach for discovering potential trait-related pathways or genes that could be transformed into molecular markers. Importantly, the application of molecular markers in breeding is multifaceted and includes applications in linkage map construction, trait-related gene localization, quantitative trait analysis, germplasm diversity analysis, genetic relationship analysis, molecular marker-assisted selection, and the detection and identification of seed purity and vigor.

### 5.1. Genetic Diversity and Evaluation and Cucumber Germplasm Selection

Genetic variation is the basis of variety improvement. The analysis of genetic diversity, species evolution, and kinship is conducive to the collection, conservation, and effective utilization of germplasm resources. Moreover, such analysis is important for determining the degree of kinship between breeding parents, which is the basis for parent selection and heterosis prediction. Molecular identification technology was used to evaluate the genetic relationships, parthenocarpy, disease resistance, and stress resistance of cucumber germplasm resources, which resulted in the identification of germplasms with desirable characteristics. Genetic diversity analysis of 280 cucumber accessions collected from four continents (Asia, Europe, America, and Africa) by the National Agrobiodiversity Center of the Rural Development Administration in South Korea and 20 commercial Korean F_1_ hybrids revealed that the accessions generally formed four subpopulations or clusters that corresponded to their geographical origins [104]. The genetic variation, marker attributes, and population structure of 104 cucumber genotypes were assessed using 23 SSR primer pairs. The information obtained would favor the selection of cucumber genotypes with high genetic diversity [22].

### 5.2. Gene Mapping

The locations of important genes can provide help for molecular marker-assisted breeding and variety improvement, and enable further cloning of target genes and gene transfer. For example, a single recessive gene that was predicted to control white immature fruit color was mapped to the distal region of cucumber chromosome 3 using SSR markers. Subsequently, 1655 homozygotes derived from 7304 F_2_ specimens of a cross of the Q1 × H4 hybrid were used for fine mapping of the white immature fruit color gene. The gene was mapped to a 100.3-kb region between markers Q138 and Q193 [105]. Based on fine mapping, BSA, and genotyping of a large F_2_ population using a kbioscience allele-specific polymorphism (KASP) assay, a candidate gene responsible for male sterility (*ms-3*) was identified and mapped to a 76-kb genomic DNA region [106].

### 5.3. Molecular Marker-Assisted Selection

The correct selection and effective separation of target traits are key to successful plant breeding. Traditional breeding methods rely mainly on phenotype selection. However, many important traits are strongly affected by environmental conditions. Molecular-marker-assisted breeding selects breeding materials at the molecular level and can be used to detect and track single or multiple genes linked to target traits, thereby reducing the blindness of breeding selection to improve breeding efficiency. Cucumber vein yellowing virus causes economic losses to cucumber crops in Mediterranean countries, some parts of India, and Africa. With the aid of genomics and BSA, SNP markers capable of selecting *cucumber vein yellowing-1 (CsCvy-1)* in different backgrounds have been identified [107]. Fruit shape is commonly modulated by both genetic and environmental factors. Chromosome segment substitution lines have been widely used to identify and map QTLs associated with target traits. In a recent study, a set of chromosome segment substitution lines, which were developed from a cross between “RNS7” (a round-fruit line) and “CNS21” (a long-stick-fruit line), was established. A set of 114 indel markers that were widely distributed across the cucumber genome were used to screen 21 QTLs for fruit shape traits. This work contributed to subsequent research on cucumber fruit shape [108].

## 6. Prospects

Genetic breeding of traditional cucumber is generally accomplished by crossbreeding, grafting, and other methods. Even though traditional breeding methods can be used to develop novel cucumber varieties, the process cannot efficiently address yield increases, disease resistance, and overall quality. With recent increases in genomic resources, there is a general trend to construct a cucumber genetic map with high saturation, practicality, and versatility. Moreover, cucumber breeding is inseparable from the combination of agronomic traits and functional genes. In the future, breeders could, on the one hand, comprehensively use genetics, multi-omics, molecular biology, and bioinformatics to identify key regulatory genes involved in related pathways and perform related functional verification to accelerate the breeding process; and on the other hand, use a variety of molecular marker technologies to construct a dense genetic linkage map, assist in trait screening, perform multi-character simultaneous marking and screening, give full play to the role of molecular markers in cucumber breeding, and improve the efficiency of cucumber breeding. In particular, breeders have discovered that genome-editing technology can be used to accurately modify specific trait-related genes, and to circumvent the introduction of redundant genes due to linkage burden. Therefore, in future, cucumber breeding could combine omics technology with genome-editing technologies such as CRISPR/Cas9 to greatly shorten the cucumber breeding cycle.

## Data Availability

Not applicable.
